# Improving chemical disease relation extraction with rich features and weakly labeled data

**DOI:** 10.1186/s13321-016-0165-z

**Published:** 2016-10-07

**Authors:** Yifan Peng, Chih-Hsuan Wei, Zhiyong Lu

**Affiliations:** 1 National Center for Biotechnology Information, Bethesda, MD 20894 USA; 2 Computer and Information Sciences, University of Delaware, Newark, DE 19716 USA

**Keywords:** Chemical-induced disease, Relation extraction, BioNLP, Text mining

## Abstract

**Background:**

Due to the importance of identifying relations between chemicals and diseases for new drug discovery and improving chemical safety, there has been a growing interest in developing automatic relation extraction systems for capturing these relations from the rich and rapid-growing biomedical literature. In this work we aim to build on current advances in named entity recognition and a recent BioCreative effort to further improve the state of the art in biomedical relation extraction, in particular for the chemical-induced disease (CID) relations.

**Results:**

We propose a rich-feature approach with Support Vector Machine to aid in the extraction of CIDs from PubMed articles. Our feature vector includes novel statistical features, linguistic knowledge, and domain resources. We also incorporate the output of a rule-based system as features, thus combining the advantages of rule- and machine learning-based systems. Furthermore, we augment our approach with automatically generated labeled text from an existing knowledge base to improve performance without additional cost for corpus construction. To evaluate our system, we perform experiments on the human-annotated BioCreative V benchmarking dataset and compare with previous results. When trained using only BioCreative V training and development sets, our system achieves an F-score of 57.51 %, which already compares favorably to previous methods. Our system performance was further improved to 61.01 % in F-score when augmented with additional automatically generated weakly labeled data.

**Conclusions:**

Our text-mining approach demonstrates state-of-the-art performance in disease-chemical relation extraction. More importantly, this work exemplifies the use of (freely available) curated document-level annotations in existing biomedical databases, which are largely overlooked in text-mining system development.

## Background

Drug/chemical discovery is a complex and time-consuming process that often leads to undesired side effects or toxicity [13]. To reduce risk and the development time, there has been considerable interest in identifying chemical-induced disease (CID) relations between existing chemicals and disease phenotypes by computational methods. Such efforts are important not only for improving chemical safety but also for informing potential relationships between chemicals and pathologies [53]. Much of the knowledge regarding known adverse drug effects or associated chemical-induced disease (CID) relations is buried in the biomedical literature. To make such information available to computational methods, several databases in life sciences such as the Comparative Toxicogenomics Database (CTD) have begun curating important relations manually [9]. However, with limited resources, it is difficult for individual databases to keep up with the rapidly-growing biomedical literature [4].

**Fig. 1:**
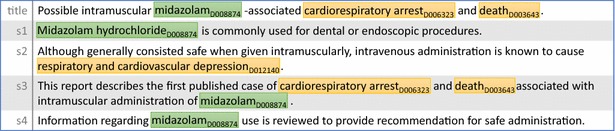
The title and abstract of a sample document (PMID 2375138). Chemical and disease mentions are marked in *green* and *yellow* respectively. <D008874, D012140> and <D008874, D006323> are two CID relation pairs

Automatic text-mining tools have been proposed to assist the manual creation [34, 45, 54] and/or to directly generate large-scale results for computational purposes [47, 49]. We recently held a formal evaluation event through the BioCreative V challenge (BC5 hereafter) to specifically assess the advances in text mining for extracting chemical-disease relations [53]. Different from previous relation extraction tasks such as protein–protein interaction, disease-gene association, and miRNA-gene interaction [23, 25, 28–32, 44], the BC5 task requires the output of extracted relations with entities normalized to a controlled vocabulary (the National Library of Medicine’s Medical Subject Headings (MeSH) identifiers were used). Furthermore, one should extract such a list of <Chemical ID, Disease ID> pairs from the entire PubMed document and many relations may be described across sentences [53]. For instance, Fig. 1 shows the title and abstract of a document (PMID 2375138) with two CID relations <D008874, D006323> and <D008874, D012140>. While the former relation (“midazolam” and “cardiorespiratory arrest”) is in the same sentence, the latter relation (“midazolam” and “respiratory and cardiovascular depression”) is not. Moreover, not all pairs of chemicals and diseases are involved in a CID relation. For instance, there is no relation between “midazolam” and “death” in Fig. 1 because the task guidelines consider “death” to be too general.

During the BioCreative V challenge, a new gold-standard data set was created for system development and evaluation, including manual annotations of chemicals, diseases and their CID relations in 1500 PubMed articles [30]. A large number of international teams participated and achieved the best performance of 57.07 in F-score for the CID relation extraction task. In this work, we aim to improve the best results obtained in the challenge by combining a rich-feature machine learning approach with additional training data obtained without additional annotation cost from existing entries in curated databases. We demonstrate the feasibility of converting the abundant manual annotations in biomedical databases into labeled instances that can be readily used by supervised machine-learning algorithms. Our work therefore joins a few other studies in demonstrating the use of the curated knowledge freely available in biomedical databases for assisting text-mining tasks [17, 46, 48].

More specifically, we formulate the relation extraction task as a classification task on chemical-disease pairs. Our classification model is based on Support Vector Machine (SVM). It uses a set of rich features that combine the advantages of rule-based and statistical methods.

While relation extraction tasks were first tackled using simple methods such as co-occurrence, lately more advanced machine learning systems have been investigated due to the increasing availability of annotated corpora [52]. Typically, the relation extraction task has been considered as a classification problem. For each pair, useful information from NLP tools including part-of-speech taggers, full parsers, and dependency parsers were extracted as features [20, 56]. In the BioCreative V, several machine learning models have been explored for the CID task, including Naïve Bayes [30], maximum entropy [14, 19], logistic regression [21], and support vector machine (SVM). In general, the use of SVM has achieved better performance [53]. One of the highest-performing systems was proposed by Xu et al. [55] with two independent SVM models, sentence-level and document-level classifiers for the CID task. We instead combined the feature vector on both the sentence and document level and developed a unified model. We believe our system is more robust and can be used more easily for other relation extraction tasks with less effort needed for domain adaptation.

SVM-based systems using rich features have been previously studied in biomedical relation extraction [5, 50, 51]. Most useful feature sets include lexical information and various linguistic/semantic parser outputs [1, 2, 15, 23, 38]. Built upon these studies, our rich feature sets include both lexical/syntactic features as previously suggested as well as task specific ones like the CID patterns and domain knowledge as mentioned below.

Although machine learning-based approaches have achieved the highest results, some rule-based and hybrid systems [22, 33] showed highly competitive results during the BioCreative Challenge. In our system, we also integrate the output of a pattern matching subsystem in our feature vector. Thus, our approach can benefit from both machine-learning and rule-based approaches.

To improve the performance, many systems also use external knowledge from both domain specific (e.g., SIDER2, MedDAR, UMLS) and general (e.g. Wikipedia) resources [7, 18, 22, 42]. We incorporate some of these types of knowledge in the feature vector as well.

Another major novelty of this work lies in our creation of additional training data from existing document-level annotations in a curated knowledge base to improve the system performance and to reduce the effort of manual text corpus annotation. Specifically, we make use of previously curated data in CTD as additional training data. Unlike the fully annotated BC5 corpus, these additional training data are weakly labeled: CID relations are linked to the source articles in PubMed (i.e. document-level annotations) but the actual appearances of the disease and chemicals in the relation are not labeled in the article (i.e. mention-level annotations are absent). Hence they are not directly applicable and have to be repurposed when used for training our machine-learning algorithm. Supervised machine-learning approaches require annotated training data which may be difficult to obtain in large scale. To acquire training data, people have recently studied various methods using unlabeled or weakly labeled data [6, 37, 48, 57, 58]. However, such data is often too diverse and noisy to result in high performance [43]. In this paper, we created our labeled data using the idea of distant supervision [37] but limit the data to be the weakly labeled article that was the source of the curated relation. Thus, this work is most closely related to Ravikumar et al. [46] with regards to creating training data using existing database curation. However unlike them, we label relations both within and across sentence boundaries and use additionally labeled data only to supplement the gold-standard corpus.

Through benchmarking experiments, we show that our proposed method already achieves favorable results to the best performing teams in the recent BioCreative Challenge when using only the gold-standard human annotations in BC5. Moreover, our system can further improve its performance significantly when incorporating additional training data, by taking advantage of existing database curation at no additional annotation cost.

## Methods

### Data

As shown in Table 1, the manually annotated BC5 corpus consists of separate training, development, and test sets. Each set contains 500 PubMed articles with their title and abstracts. All chemical and disease text mentions and their corresponding concept IDs (in MeSH) were provided by expert annotators. The CID relations were annotated at the document level.

**Table 1:** Statistics of the corpora

Corpus	Documents	CID Pairs	Unique
BC5 training	500	1038	927
BC5 development	500	1012	887
BC5 test	500	1066	941
CTD-Pfizer	18,410	33,224	15,439

Besides the (limited) manual annotation data sets, we created additional training data from existing curated data in the CTD-Pfizer collaboration [10] where the raw data contains 88,000 articles with document-level annotations of drug-disease and drug-phenotype interactions. To make this corpus consistent with the BC5 corpus, we first filtered those without CID relations in the title/abstracts as some asserted relations are only in the full text. Moreover, the raw data contains no mention-level chemical and disease annotations. Thus, we applied two state-of-the-art bio-entity taggers tmChem [27] and DNorm [26] to recognize and normalize chemicals and diseases respectively. To maximize recall, we also applied a dictionary look-up method with a controlled vocabulary (MeSH). As a result, we obtained 18,410 abstracts with 33,224 CID relations and made sure they have no overlap with the BC5 gold standard.

### Method

**Fig. 2:**
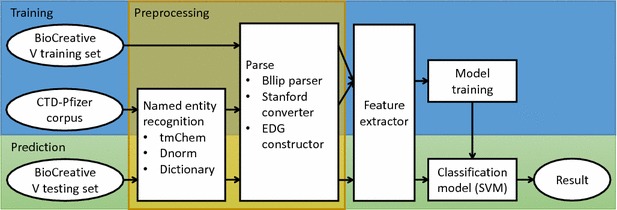
The pipeline of our CID extraction system

We formulated the chemical-disease relation extraction task as a classification problem that judges whether a given pair of chemical and disease was asserted with an induction relation in the article. Figure 2 shows the overall pipeline of our proposed CID extraction system using machine learning.

We treat the CID task as a binary classification problem. In the training step, we construct the labeled feature instances from the training set (BC5 training set and CTD-Pfizer corpus). For the BC5 training set, we use the gold-standard entity annotations. For the CTD-Pfizer corpus, we use the recognized chemical and disease mentions as described in previous section. To maximize recall, we also applied a dictionary look-up method with a controlled vocabulary (MeSH). Following name detection, we split the raw text into individual sentences by Stanford sentence splitter [35], and obtain the parse trees using Charniak–Johnson parser with McClosky’s biomedical model [8, 36]. We then apply the Stanford conversion tool with the “CCProcessed” setting [12] and the construction method described in Peng et al. [41] to obtain the extended dependence graph (EDG). In the feature extractor module, for each pair of <Chemical ID, Disease ID> in one document, we iterate through all mention pairs to extract mention-level features. We then merge these mention-level features and add ID-level features to acquire the final feature vector between <Chemical ID, Disease ID>. Finally, Support Vector Machine (SVM) is applied to obtain the model.

In the prediction step, we use the same pipeline to construct the unlabeled feature instances from the BC5 test set, then predict their classes (i.e. whether there is a CID relationship) using the learned model.

In the following subsections, we explain both lexical and knowledge-based features.

### Bag-of-words features

**Fig. 3:**
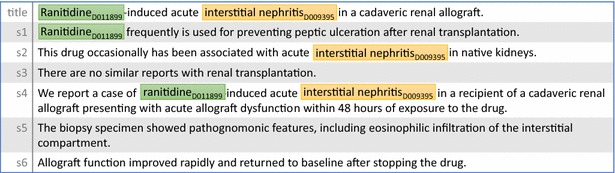
The title and abstract of a sample document (PMID 11431197). Chemical and disease mentions are marked in *green* and *yellow* respectively. <D011899, D009395> is a CID relation pair

The Bag-of-Words (BOW) features include the lemma form of words around both chemical and disease mentions and their frequencies in the document. Different types of named entity mentions have the same BOW feature set. In our system, we take the context of both chemical and disease mentions into account using a window of the size of 5. Therefore, the mention itself and two words before and after are extracted. We do not allow the window to slide across the sentence boundary, but two windows can be in two sentences where the chemical and disease are mentioned respectively. As an example, the BOW features of “D011899” in Fig. 3 are “induce”, “acute”, “frequently”, “is”, “case”, and “of”. Note that “induce” and “acute” appear twice (line 1 and 5).

### Bag-of-Ngram features

The Bag-of-Ngram (BON) features are pairs of consecutive lemma form of words from chemical to disease (or vice versa) when both are in the same sentence. These features (also called N-gram language model features) enrich the BOW feature by word phrases, hence can store the local context. For example, the bag-of-bigram features of Fig. 3 are “(D011899, induce)”, “(induce, acute)” and “(acute, D009395)”. In our system, we use unigrams, bigrams and trigrams. In other words, BON has a sliding window size of 1, 2, and 3 respectively. Please note that we use MeSH IDs instead of actual Chemicals or Diseases in the BON features because MeSH ID is able to differentiate different types of chemicals and diseases thus achieving better results in our experiments.

### Patterns

A common approach to relation extraction involves manually developing rules or patterns, which usually achieves a high precision but is sometimes criticized for its low recall. In our system, we use the output of rule matching as features. It gives the feature vector of *four* dimensions as its output, each of which corresponds to one trigger in matched patterns: “cause”, “induce”, “associate”, or “produce”.

In this paper, we use the Extended Dependency Graph (EDG) to represent the structure of the sentence [41]. The vertices in an EDG are labeled with information such as the text, part-of-speech, lemma, and named entity, including chemical and diseases. EDG has two types of dependencies: syntactic dependencies and numbered arguments. The syntactic dependencies are obtained by applying Stanford dependencies converter [12] on a parse tree obtained by the Bllip parser [8]; the numbered arguments are obtained by investigating the thematic relations described by verbal and nominal predicates. In this paper, we use “arg0” for the agent and “arg1” for other roles such as patient and theme.

**Fig. 4:**
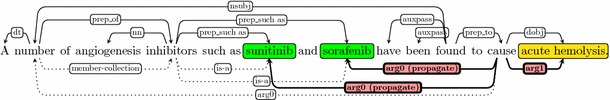
The Extended Dependency Graph of the sentence “A number of angiogenesis inhibitors such as sunitinib and sorafenib have been found to cause acute hemolysis” (PMID: 20698227)

Figure 4 demonstrates an EDG of a sentence. Edges above the sentence are Stanford dependencies, and edges below are newly created numbered arguments. “arg0” is a numbered-argument that unifies the realization of active, passive, and nominalized forms of a verb (“cause”) with its argument (“number”). “member-collection” links a generic reference (“number”) to a group of entity mentions (“inhibitors”). “is-a” indicates the relation between X (“sunitinib” and “sorafenib”) and Y (“inhibitors”) when X is a subtype of Y.

Note that the original “arg0” links “cause” to “number”, but “number” is not a named entity. To find the real target of “arg0”, EDG introduces several semantic edges such as “member-collection” and “is-a” (as shown in dotted edges below the sentence). Then EDG propagates “arg0” from “number” to “sunitinib” and “sorafenib” using the following rule. For more details of the way EDG is constructed, please refer to Peng et al. [41].

**Table:** 

*arg0* (cause, number) *member*-*collection*(number, inhibitors) *is*-*a* (sunitinib, inhibitors) *is*-*a* (sorafenib, inhibitors)	⇒	*arg0* (cause, sunitinib) *arg0* (cause, sorafenib)

**Fig. 5:**
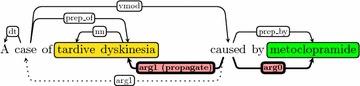
The Extended Dependency Graph of the text “A case of tardive dyskinesia caused by metoclopramide” (PMID: 6727060)

Oftentimes, “arg0” or “arg1” links the head word of a phrase that is not a chemical or disease. For example, in “A case of tardive dyskinesia caused by metoclopramide” (Fig. 5), “arg1” links “cause” to “case” but not “tardive dyskinesia”. In such cases, we skip the head word by propagating “arg1” (or “arg2”) from “case” to “tardive dyskinesia”. This idea is based on the notion of a core-term proposed by Fukuda et al. [16], Narayanaswamy et al. [39] and the method of conjunction propagation in De Marneffe and Manning [11]. “arg1 (propagate)” in Fig. 5 serves this purpose.

EDG is able to unify different syntactic variations in the text, thus only one rule is used in our system to extract CID. “Chemical ← arg0 ← trigger → arg1 → Disease”, where the “trigger” is one of the four words: “cause”, “induce”, “associate”, or “produce”. For each mention pair, the rule-based system will output four Boolean values indicating whether a rule can be applied. We incorporate these four values in the feature vector.

### Shortest path features

The shortest path features include v-walks (two lemmas and their directed link) and e-walks (a lemma and its two directed links) on EDG when two mentions are in the same sentence [24]. But unlike [24], which does not include link directions, we include the link directions in v-walks and e-walks. Table 2 illustrates the shortest path between the pair in Figs. 3 and 5. Note that although sentences in both figures have different surface word sequences, they share the same semantic structure (numbered arguments) in EDG. Thus, their shortest paths (and v-walks and e-walks) are the same. This characteristic is helpful to generalize machine learning methods more easily.

**Table 2:** Shortest path, v-walks, and e-walks of sample sentences in Figs. 4 and 5

Shortest path	Chemical ← arg0 ← cause → arg1 → Disease
v-walks	cause → arg0 → Chemical
	cause → arg1 → Disease
e-walks	arg0 ← cause → arg1

We also take into account the length of the shortest path by introducing *λ* ^*length*^, where 0 < λ ≤ 1 and *length* is the length of the shortest path. This feature down-weights the contribution of the shortest path exponentially with its lengths. If there are multiple shortest paths between the chemical and disease (in the same sentence or across multiple sentences), we extract all v-walks and e-walks and average *λ* ^*length*^. In this paper, we adjust *λ* to 0.9 based on previous experience [1, 2].

### Statistical features

We also extracted statistical features shown in Table 3. For Boolean features, we merged mention-level features by using the “or” operation. For numerical features, we averaged mention-level features. Overall speaking, these ad-hoc features were included to capture the importance of a chemical/disease in an article (#1–#8), the strength between a possible disease and chemical relation (#9–#11), and the context that a disease or chemical is involved in CID relations (#12–#19). It is noteworthy that for the 10th and 11th features, we only check the existence of a target relation pair in CTD or MeSH but not the actual curated articles in either resource.

**Table 3:** Statistical features

Feature		Type
1	# of chemical mention	Numeric
2	# of disease mention	Numeric
3	Is chemical in title	Boolean
4	Is disease in title	Boolean
5	Is chemical in the 1st sentence of the abstract	Boolean
6	Is disease in the 1st sentence of the abstract	Boolean
7	Is chemical in the last sentence of the abstract	Boolean
8	Is disease in the last sentence of the abstract	Boolean
9	Are both of chemical and disease in the same sentence	Boolean
10	Is disease-chemical relation curated by CTD in the past	Boolean
11	Do both disease and chemical exist in the MeSH indexing in the past?	Boolean
12	Is any keyword around the disease, such as therapy, complicating, affect, etc.	Boolean
13	Is any keyword around the chemical, such as 3.0 mEg/L, mg, etc.	Boolean
14	Is “increase” or “decrease” around chemical	Boolean
15	Is “increase” or “decrease” around disease	Boolean
16	Is “*p* value” around chemical	Boolean
17	Is “p-value” around disease	Boolean
18	Is “men”, “women”, or “patient” around chemical	Boolean
19	Is “men”, “women”, or “patient” around disease	Boolean

## Results and discussion

### Results

We report our system performance in two scenarios: with or without using the human-annotated entity mentions. First we evaluated our relation extraction system over text-mined mentions. This gave the real-world performance of our end-to-end system and enabled direct comparisons to others’ work. Second, to help identify errors due to entity recognition, we also evaluated our system using the manual entity annotations of chemicals and diseases in the BC5 test set. Table 4 shows the named entity recognition results on the BC5 test set. Using tmChem and DNorm (trained on the BC5 training and development data) respectively, we achieved F-scores of 79.94 and 90.49 %, respectively.

**Table 4:** Evaluation of named entity results in normalized concept identifiers

Named entity	Precision	Recall	F-score
Disease concepts	78.77	81.14	79.94
Chemical concepts	88.49	92.57	90.49

Table 5 shows the CID results on the BC5 test set using gold, as well as text-mined mentions. The gold mentions are provided in the BC5 test set, and the text-mined mentions were computed via tmChem [27] and DNorm [26] for chemicals and diseases respectively. In both cases, we consider all possible chemical-disease pairs in an abstract and then used our machine-learning model to classify if a given pair holds a CID relation. Performance is measured by the standard precision, recall, and F-score. For comparison purposes, we also include the average and best team results in BioCreative 5 CID task, as well as a baseline result using entity co-occurrence [53]. For our own system, we show the system performance with an incremental change of the training data. We first used only the BC5 training set (row 1). By combining BC5 human-annotated training and development dataset, we obtained an F-score of 57.51 % (row 2), which is significantly better than the baseline or the average team results [53] and compares favorably to the best results during the recent BioCreative challenge [55]. Then, we added more automatically-labeled training data, randomly selected from the CTD database, in succession (rows 3–6). We achieved the highest performance of 61.01 % in F-score when the entire set of 18,410 articles was added for training.

**Table 5:** Evaluation of CID results

Team/training corpus	Using text-mined entity mentions			Using gold entity mentions		
	Precision	Recall	F-score	Precision	Recall	F-score
Co-occurrence baseline	16.43	76.45	27.05			
Avg team results	47.09	42.61	43.37	–	–	–
Best team results	55.67	58.44	57.03	–	–	–
1. Train	51.55	59.19	55.11	62.07	64.17	63.10
2. Train + dev	64.24	52.06	57.51	68.15	66.04	67.08
3. Train + dev + 1000	63.78	53.85	58.39	68.12	68.95	68.53
4. Train + dev + 5000	62.50	56.75	59.49	67.63	72.33	69.90
5. Train + dev + 10,000	64.49	56.57	60.27	69.64	71.86	70.73
6. Train + dev + 18,410	65.59	56.94	61.01	71.07	72.61	71.83

### Contribution of features

Table 6 compares the effects of different features. Row 1 shows the performance using all features. Then we removed each feature set in turn and retrained the model. In these feature-ablation experiments, only BC5 task data were used and the performance was measured based on text-mined entities.

**Table 6:** Contributions of different features

	Features	Precision (%)	Recall (%)	F-value (%)	F-value change (%)
1	All features	64.24	52.06	57.51	
2	- BOW	63.09	51.31	56.60	−0.91
3	- BOB	61.24	52.63	56.61	−0.90
4	- Pattern	61.83	51.22	56.03	−1.48
5	- Shortest path	62.03	52.72	57.00	−0.51
6	- Statistical	53.29	41.74	46.82	−10.69
7	- #1 ~ #8	62.54	50.75	56.03	−1.48
8	- #1 and #2	62.90	51.69	56.75	−0.76
9	- #3 and #4	63.31	51.97	57.08	−0.43
10	- #5 ~ #8	63.23	51.78	56.94	−0.57
11	- #9 ~ #11	54.04	45.12	49.18	−8.33
12	- #9	63.62	52.16	57.32	−0.19
13	- #10	57.09	45.31	50.52	−6.99
14	- #11	61.49	50.47	55.44	−2.07
15	- #12 ~ #19	63.79	52.06	57.33	−0.18

The most significant performance drop occurred when the set of statistical features (−10.69) was removed. In particular, the features checking relation existence in curated databases are quite informative. The second major decrease in performance is due to the removal of EDG with numbered arguments (−1.48 for pattern and −0.51 for shortest path). On the other hand, removing those contextual features #12 ~ #19 from the statistical set did not significantly reduce the performance. It is possible that other features such as BOW, BOB, and shortest path have already captured the context information.

It is also noteworthy that by removing patterns, the precision of the system decreased 2.4 % (from 64.24 to 61.83), while the recall stayed almost the same (0.8 %). This provides support for the usefulness of pattern matching in our system.

Only one pattern (“Chemical ← arg0 ← trigger → arg1 → Disease”) was used. Overall, this simple pattern can achieve a high precision of 73.11 % (Table 7). At the same time, we observed the need to experiment more patterns in the next step.

**Table 7:** Precision on BC5 training set

Trigger	TP	FP	Precision (%)
Associate	29	9	76.32
Cause	21	10	67.74
Induce	179	65	73.36
Produce	12	4	75.00
Total	242	89	73.11

### Error analysis

We show in Table 5 the highest performance of CID relation extraction using the BC5 test set. First, we would like to compare our performance to the inter-annotator agreement (IAA), which generally indicates how difficult the task is for humans and is often regarded as the upper performance ceiling for automatic methods. Unfortunately, the CID relations in the BC5 test set were not double annotated thus the IAA scores by expert annotators are not available for comparison. Alternatively, we compared our performance to the agreement scores from a group of non-experts where IAAs of 64.70 and 58.7 % were obtained respectively, with the use of gold or text-mined entities. As can be seen from Table 5, our system performance of 71.87 and 61.01 % in F-scores compare favorably in both scenarios.

Compared with other relation extraction tasks (such as PPI), we believe CID benefited from two main factors: a) the BioCreative V task provided larger task data which included not only document-level annotations but also mention-level annotations, which are not available in many other similar tasks; and b) the recent advances in disease and chemical named entity recognition and normalization. In fact, the automatic NER and normalization performance for disease and chemicals are approaching human IAAs (F-score in the 80 and 90s, respectively). Unfortunately, this is still not the case for other entities such as gene and proteins.

**Fig. 6:**
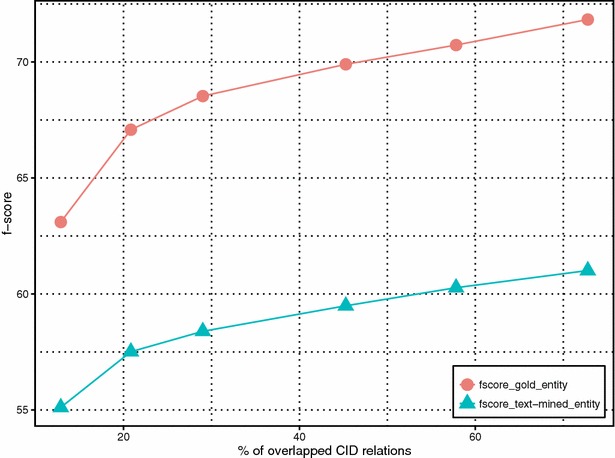
The relationship between the percentage of overlapped CID relations and the method performance in F-scores with (fscore_gold_entity) and without (fscore_text-mined_entity) using gold entities

**Fig. 7:**
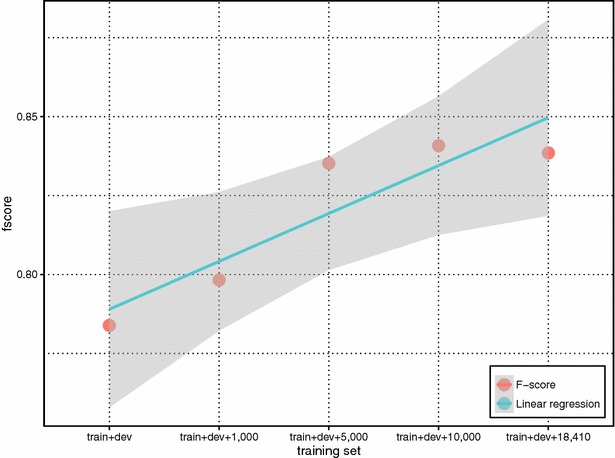
The performance changes of the overlapped CID relations in the test set

**Fig. 8:**
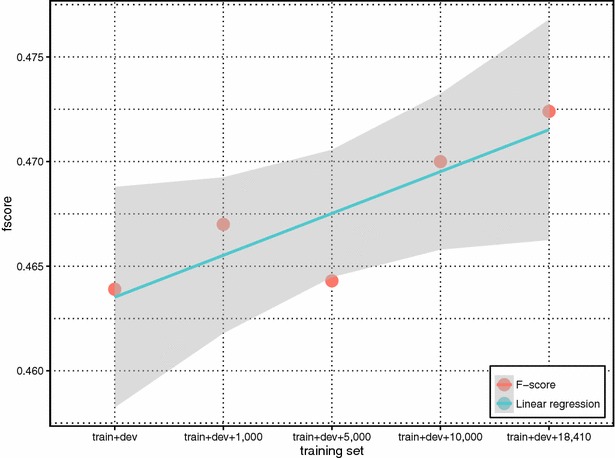
The performance changes of non-overlapped CID relations in the test set

From Table 5, our results show strong performance boost from using the weakly labeled training data. Despite noisiness, such data can significantly increase the coverage of unique chemical-disease relations in the test data set. Indeed, the overlap of unique chemical-disease relations between the union of training and development sets (train + dev) and test set are 196 relations (20.8 % of unique CIDs in the test set). But after adding additional data, the overlap increases to 685 relations, covering 72.8 % of CIDs in the test set. Figure 6 shows the relationship between the percentage of overlap and our method performance in F-scores with (fscore_gold_entity) and without using gold entities (fscore_text-mined_entity). It is clear that more curation data, despite the fact that they are not annotated for training machine-learning purposes, helps improve the coverage and system performance. We further separated CID relations in the test set into two groups with respect to whether a given relation appeared in the training set (i.e. overlapping or not). Figures 7 and 8 show the f-score changes in each group with additional data and demonstrate that both groups benefited from adding more weakly labeled data to the training set with more performance gains in the first “overlapping” group.

Comparing the results with and without using gold-standard mentions in the test set (row 6 in Table 5), our results indicate that errors by the named entity tagger bring 10.8 % decrease in F-score for the CID extraction.

We further analyzed the errors made by our system on the BC5 test set using text-mined entity mentions (Table 8). About 40 % of the total errors in CID relations were because of incorrect NER or normalization. Take a false negative error as an example, in “In spite of the fact that TSPA is a useful IT agent, its combination with MTX, ara-C and radiotherapy could cause severe neurotoxicity” (PMID 2131034), “TSPA” was recognized a chemical mention but was not correctly normalized to the MESH ID D013852.

**Table 8:** Statistics of extraction errors by our method

Error type	FN	FP	Total	%
NER or normalization errors	254	58	312	39.90
CID relations mentioned in single sentences	148	124	272	34.78
CID relations asserted across sentences	63	54	117	14.96
Extracted disease or chemical in CID is too general	0	46	46	5.88
The extracted disease/chemical pair is a treatment relation	0	29	29	3.71
Annotated CID relations absent in the abstract	6	0	6	0.77
Total	471	311	782	

Besides NER errors, nearly 35 % of incorrect results were extracted in single sentences. For example, our method failed to extract the CID relation of “renal injury” (MeSH: D058186) and “diclofenac” (MeSH: D004008) from the following sentence: “The renal injury was probably aggravated by the concomitant intake of a non-steroidal anti-inflammatory drug, diclofenac”. Our pattern feature could not be extracted because “aggravate” is not one of our relation trigger words. In addition, the mixture of chemical-induced disease and chemical-treated disease relations within one sentence often poses extra challenges for feature/pattern extraction. Finally, 15 % of total errors were CID relations that are asserted across sentence boundaries, which motivates us to investigate how to capture long-distance CID relations in the future.

## Conclusions

In conclusion, this paper discusses a machine-learning based system to successfully extract CID relations from PubMed articles. It may be challenging to directly apply our method to full-length articles (because considerable time may be required to process linguistic analyses) or abbreviated social media text [3, 40]. Another limitation is related to the NER errors: we can expect relation results to increase when mention-level NER results are further improved. In the future, we also plan to investigate the robustness and generalizability of our core approach to other types of important biomedical relations.
